# When Roads Remove Trees, Conservation Must Move Fast: A Rescue Translocation Model for Keralam’s Native Epiphytic Orchids

**DOI:** 10.3390/plants15203153

**Published:** 2026-10-15

**Authors:** Mithun Venugopal, Pradeep N. Sukumaran, Viswambharan Sarasan

**Affiliations:** 1 KSCSTE-Malabar Botanical Garden and Institute for Plant Sciences, Kozhikode 673014, Keralam, India; mithunv233@gmail.com (M.V.); drnspradeep@gmail.com (P.N.S.); 2 Royal Botanic Gardens, Kew, Richmond, Surrey TW9 3AE, UK

**Keywords:** orchid conservation, epiphytes, phorophyte, conservation translocation, urban biodiversity, coastal stress, salt spray, Keralam, Kozhikode, nature connection

## Abstract

Development-related tree removal can immediately eliminate epiphytic orchids and specialised canopy habitats, yet evidence for rescue translocation remains limited. We assessed 27,324 mature native orchids representing 21 species, rescued during road widening in Keralam, India, and translocated to three contrasting recipient landscapes. Of the 27,324 translocated orchids, 19,511 survived (71.4%) after 12–24 months. Among the surviving plants, 5224 flowered (26.8%), of which 1934 produced capsules (37.0%). Recovery differed among recipient sites, orchid species and phorophytes. The buffered urban site supported the highest survival, whereas the exposed coastal site imposed a strong establishment filter but supported relatively high flowering and capsule production among survivors. Regional endemic and cool-growing orchids also survived and reproduced, although establishment was lower and more site-dependent. Phorophyte identity was most strongly associated with establishment, while root growth consistently predicted survival, flowering and capsule production. Embryo-containing seeds occurred in all sampled capsule collections, but formal physiological viability and germination tests were not undertaken. Rescue translocation can therefore reduce otherwise likely losses when tree removal is unavoidable. The findings support pre-felling surveys, timely rescue, recipient site screening, testing across candidate phorophytes and staged long-term monitoring, while the operational allocation design prevents causal attribution or prescriptive species–phorophyte matching.

## 1. Introduction

Human-driven land-use change and habitat fragmentation are accelerating biodiversity loss globally, with disproportionate consequences in biodiversity hotspots, where exceptional endemism coincides with extensive historical habitat loss [1,2,3]. Epiphytic orchids are particularly vulnerable to these pressures because removal of a single mature tree can simultaneously eliminate established plants, specialised bark substrates, buffered canopy microclimates and opportunities for future recruitment. Protected areas alone may be insufficient where orchids also occupy roadside trees, plantations, and other human-modified landscapes. Nevertheless, appropriately designed and monitored conservation interventions can halt or reverse biodiversity declines [4]. Conservation planning must consequently extend into working and public landscapes that can provide complementary habitat, maintain connectivity and support species recovery alongside human use [5]. Ultimately, the scale of future biodiversity loss will depend on decisions made today, particularly those concerning land-use planning, ecological restoration and climate action [6].

The Western Ghats and their foothills in Keralam, India, are a global micro-hotspot for native orchids [7]. Keralam occupies only about 1% of India’s land area yet supports nearly 22% of the country’s native orchid flora, largely because the mountainous, high-rainfall landscapes of the Western Ghats provide exceptionally diverse habitats and microclimates. However, the state’s limited land area, dense human settlement and growing demand for housing and infrastructure leave increasingly little space for development without encroaching upon remaining natural and semi-natural habitats, placing this disproportionately rich biodiversity at substantial risk.

Orchids and other vascular epiphytes are particularly vulnerable to habitat destruction because the removal of host trees simultaneously eliminates growing substrates, canopy protection, buffered microclimates and local sources for recolonisation. Logging and fragmentation can reduce epiphyte biomass and species richness, shift communities from moisture-dependent towards drought-tolerant taxa, and restrict shade-associated orchids to buffered forest interiors [8,9,10]. In Peninsular Malaysia, surveys and rescue operations in logged and degraded forests documented 109 orchid species, including rare, endemic and previously undescribed taxa, demonstrating that trees threatened by clearance may support substantial orchid diversity that would otherwise remain undocumented and be lost [11].

Evidence from China and Southeast Asia shows that orchid declines associated with infrastructure development, forest conversion and plantation expansion are widespread across biodiversity-rich regions, but also demonstrates that rescue, translocation and habitat restoration can provide practical conservation responses. A national review from China documented 222 conservation translocation cases involving 154 plant species, many initiated in response to large-scale habitat destruction associated with hydropower development [12]. Orchid-focused assessments further recorded 55 translocation cases involving 43 species from 21 genera, encompassing nature reserve rescue, experimental reintroduction and restoration for sustainable use. Propagation-assisted recovery has also successfully established new populations of threatened epiphytic orchids in the wild [13,14,15].

Comparable pressures occur across Southeast Asia. In Sumatra, conversion of rainforest to monocultural rubber and oil palm plantations substantially altered vascular-epiphyte communities and reduced their conservation value, although structurally more complex jungle-rubber systems retained some refuge value for orchids and other epiphytes [16]. In South Vietnam, epiphytic communities developed in planted forests over approximately 2 decades but did not reproduce the composition of nearby primary forests, indicating that habitat reconstruction may support recovery without fully replacing natural forest communities [17]. Collectively, these studies demonstrate both the vulnerability of epiphytic taxa to human-driven habitat transformation and the value of integrating rescue, propagation, translocation and long-term monitoring into development and land-use planning.

Roadside trees in Keralam are important reservoirs of native epiphytic orchids, particularly in the northern districts, where many species have restricted distributions. Surveys in Wayanad recorded approximately 600 trees representing 72 tree taxa and supporting nearly 10,000 orchids from 13 species, including six regional endemics [18]. However, road construction and widening are removing mature phorophytes and their associated orchid communities [18,19].

Large-scale urban orchid restoration has been demonstrated in Singapore and South Florida, where in vitro-propagated native orchids have been planted on trees in parks and other public landscapes [20,21]. The present study differs by using mature orchids rescued from trees that had been felled or were scheduled for removal. Many were already capable of flowering, allowing early assessment of establishment, reproduction and potential changes in flowering phenology. The three geographically close but ecologically contrasting recipient sites in Kozhikode represent an urban wildlife park, a sheltered botanical garden and an exposed coastal landscape, respectively. As part of our previous studies, more than 17,000 native orchids representing 22 species were initially transferred to Sarovaram Biopark (Sarovaram) [19], with subsequent planting at KSCSTE-Malabar Botanical Garden and Institute for Plant Science (Malabar Botanical Garden) and Kappad Beach (Kappad). Of the 22 orchid species initially rescued, 21 had complete site × phorophyte outcome records and were included in the present analysis; *Oberonia proudlockii* was excluded because updated phorophyte-level records were unavailable.

The objectives of this study were to evaluate the conservation potential of orchid rescue and translocation in a rapidly changing tropical landscape, assess the establishment and reproductive performance of rescued orchids, and examine their value for public engagement and biodiversity awareness. We evaluated translocation outcomes over 12–24 months by quantifying survival, flowering and capsule production. We also tested the effects of site, orchid species, phorophyte and growth traits, and assessed the performance of regional endemic and cool-growing orchids. As a pilot study, we evaluated the germination of seeds from a single orchid species on a selected phorophyte. We also examined whether displaying rescued orchids in accessible public landscapes could support biodiversity interpretation and public engagement. The study therefore evaluates whether development-threatened orchids can be transformed into living conservation assets through biological recovery and public engagement, and whether this integrated approach could provide a scalable mitigation model for Keralam and other orchid-rich biodiversity hotspots.

## 2. Results

### 2.1. Overall Translocation Outcomes

Although all three recipient sites were located within the low-elevation coastal plain of Kozhikode, they represented contrasting ecological settings. Kappad was characterised by an exposed maritime setting with greater wind, solar and salt spray exposure; Sarovaram Bio Park comprised a humid, thermally buffered urban wetland with mangrove cover; and Malabar Botanical Garden provided a heterogeneous mosaic of marshy, shaded and moderately elevated microsites. These differences in exposure, canopy structure, atmospheric moisture and substrate-drying rates may have contributed more strongly to variation in orchid establishment than the relatively small differences in altitude (Table S1). Complete allocation, survival, flowering and capsule production outcomes for all 166 orchid species × recipient site × phorophyte combinations are provided in Table S2, Sheet 1, with the associated model-partitioning results presented in Sheets 2 and 3. Recipient site remained significantly associated with survival after accounting for orchid species composition and phorophyte identity (likelihood ratio χ^2^ = 881.61, df = 2, *p* < 0.001), indicating that unequal species allocation did not fully explain the differences among sites. Orchid species composition nevertheless contributed most strongly to survival, flowering and capsule production, while phorophyte identity also contributed independently to all three recovery stages.

Across the three recipient landscapes, 27,324 rescued orchids were translocated, comprising 21 orchid species and 26 phorophyte taxa. Overall, 19,511 plants survived (71.4%), 5224 flowered (19.1% of planted orchids; 26.8% of surviving plants), and 1934 produced capsules (7.1% of planted orchids; 37.0% of flowering orchids). Sarovaram showed the strongest establishment and the largest reproductive output (Table 1).

### 2.2. Reproductive Transition After Establishment

Flowering among established plants was highest at Kappad (35.6%), compared with Sarovaram (26.3%) and Malabar Botanical Garden (27.0%). Capsule production among flowering orchids was also highest at Kappad (62.3%), compared with Sarovaram (34.8%) and Malabar Botanical Garden (40.1%). All aggregate site comparisons were significant (Table 2).

### 2.3. Associations Between Site, Orchid Species and Phorophyte Identity, and Recovery

Likelihood ratio tests showed significant independent effects of site, orchid species and phorophyte identity on survival (Table 3), demonstrating that successful establishment depended on the recipient landscape, orchid identity and host tree (Figure S2).

### 2.4. Growth Trait Effects

Root growth was the most consistent positive predictor of recovery. Leaf length and leaf width showed response-specific effects (Table 4).

### 2.5. Performance of Regional Endemic Orchids

The regional endemic group comprised 12 species: eight strict Indian endemics and four species shared only between India (southern Western Ghats) and Sri Lanka. Regional endemics comprised eight strict Indian endemics (*Aerides crispa*, *Bulbophyllum fimbriatum*, *B. rosemarianum*, *Dendrobium barbatulum*, *D. heyneanum*, *D. nodosum*, *D. ovatum* and *Vanda sathishii*) and four India–Sri Lanka shared species (*Coelogyne breviscapa*, *Luisia birchea*, *Gastrochilus acaulis* and *Vanda wightii*)*. V. sathishii* was originally recorded as *V. thwaitesii* and *D. nodosum* as *Flickingeria nodosa*.

Across the sites, 6126 plants of regional endemic orchids were planted, 3368 survived, 510 flowered and 207 produced capsules. Survival was highest at Sarovaram (62.6%). Flowering among surviving plants was highest at Kappad (33.3%), whereas capsule production among flowering orchids was highest at Malabar Botanical Garden (43.8%). Kappad reproductive percentages were based on only 42 surviving plants and 14 flowering orchids (Table 5).

Seeds containing visible embryos were recorded in all assessed capsule collections. Observed regional endemic survival differed strongly among recipient sites (χ^2^ = 383.48, df = 2, *p* < 0.001), and flowering among surviving plants also differed significantly (χ^2^ = 11.62, df = 2, *p* = 0.003). In contrast, capsule production among flowering regional endemic orchids did not differ significantly among sites (χ^2^ = 1.48, df = 2, *p* = 0.476).

### 2.6. High-Altitude, Hill- and Mist Forest-Associated Regional Endemics

The ecologically sensitive subset comprised 3146 planted orchids, of which 1671 survived, 349 flowered and 116 produced capsules. Sarovaram supported the highest survival (56.3%), while flowering among surviving plants was higher at Malabar Botanical Garden (39.0%) and Kappad (47.6%) (Table 6).

Survival of high-altitude, hill- and mist forest-associated regional endemics differed significantly among sites (χ^2^ = 91.55, df = 2, *p* < 0.001), as did flowering among surviving plants (χ^2^ = 60.92, df = 2, *p* < 0.001). Capsule production among flowering orchids did not differ significantly among sites.

### 2.7. Associations of Recipient Site and Regional Endemic Status

To compare recovery between regional endemic and other native orchids while accounting for variation among orchid species and phorophytes, population-level probabilities were estimated from crossed binomial GLMMs for each recipient site across the three sequential recovery stages. Population-level predictions from the crossed binomial models indicated variation among recipient sites and between regional endemic and other native orchid species (Table 7). Predicted survival was highest at Sarovaram Biopark, intermediate at Malabar Botanical Garden, and lowest at Kappad Beach. Regional endemic orchids showed lower predicted survival than other native species, although the magnitude of this difference varied among sites. Predicted flowering among surviving plants also differed among recipient sites and between the two biogeographic groups. Capsule production among flowering plants showed comparatively high uncertainty, and these estimates should be interpreted cautiously because the capsule production model did not converge satisfactorily. Collectively, the results indicate that recipient site conditions were strongly associated with post-translocation performance and that regional endemic orchids may require particularly careful site selection and monitoring.

### 2.8. Phorophyte Associations

**Figure 1:**
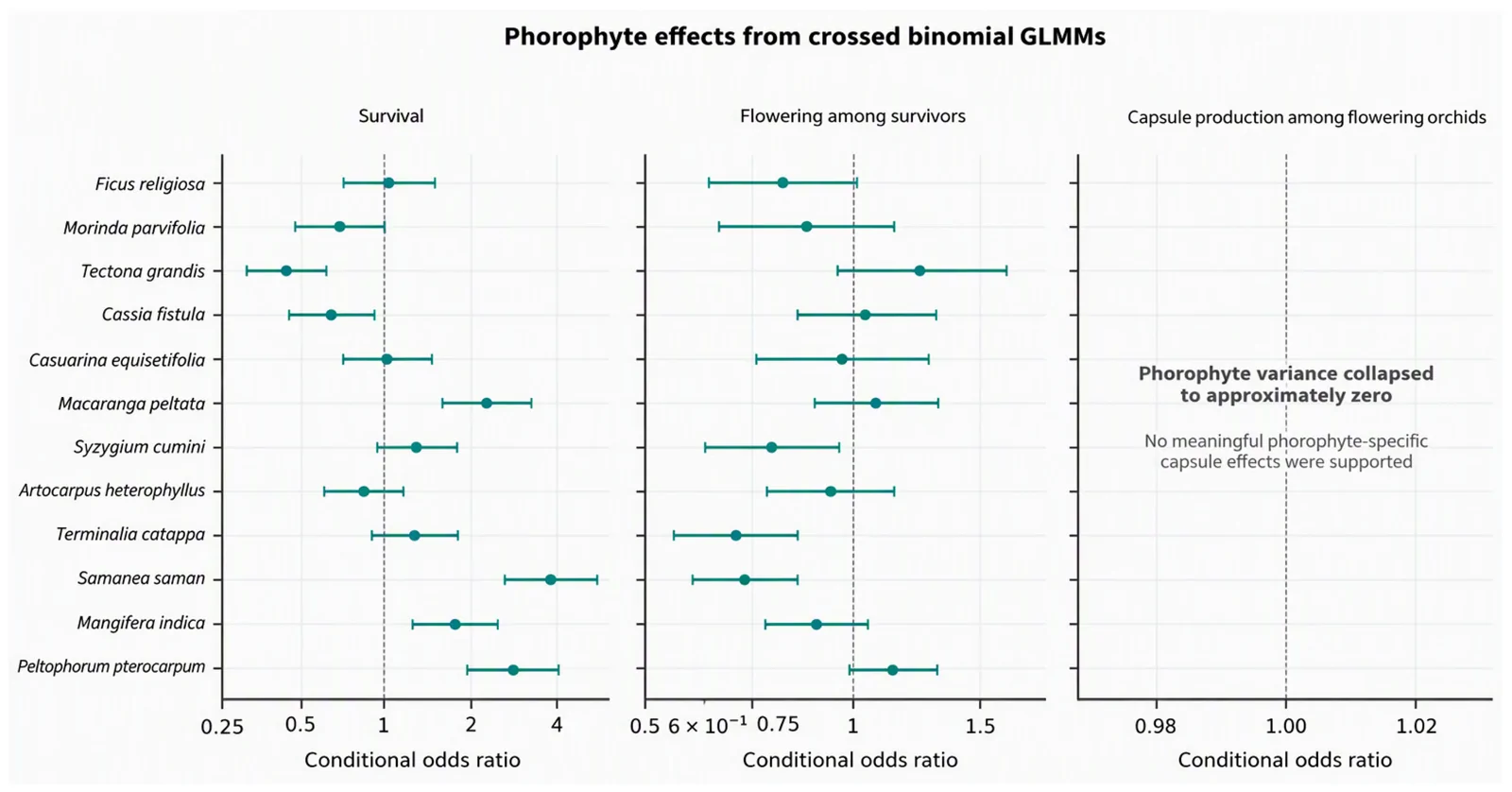
Phorophyte effects on orchid survival, flowering and capsule production following rescue translocation. Points and horizontal error bars show conditional odds ratios and 95% confidence intervals for the 12 most frequently used phorophytes. The dashed line denotes an odds ratio of 1. Models included recipient site, regional endemic status and their interaction as fixed effects, with orchid species and phorophyte identity as crossed random intercepts.

Phorophyte identity was most strongly associated with survival and more weakly associated with flowering among surviving plants (Figure 1). For capsule production among flowering orchids, the estimated phorophyte random effect variance approached zero, providing no meaningful evidence of phorophyte-specific differences after flowering. These associations should not be interpreted as species-specific compatibility rankings because allocation was operational, planting numbers were unequal, and most orchid species were not replicated across the same phorophytes within recipient sites.

### 2.9. Seed Viability and Pilot Field Sowing

**Figure 2:**
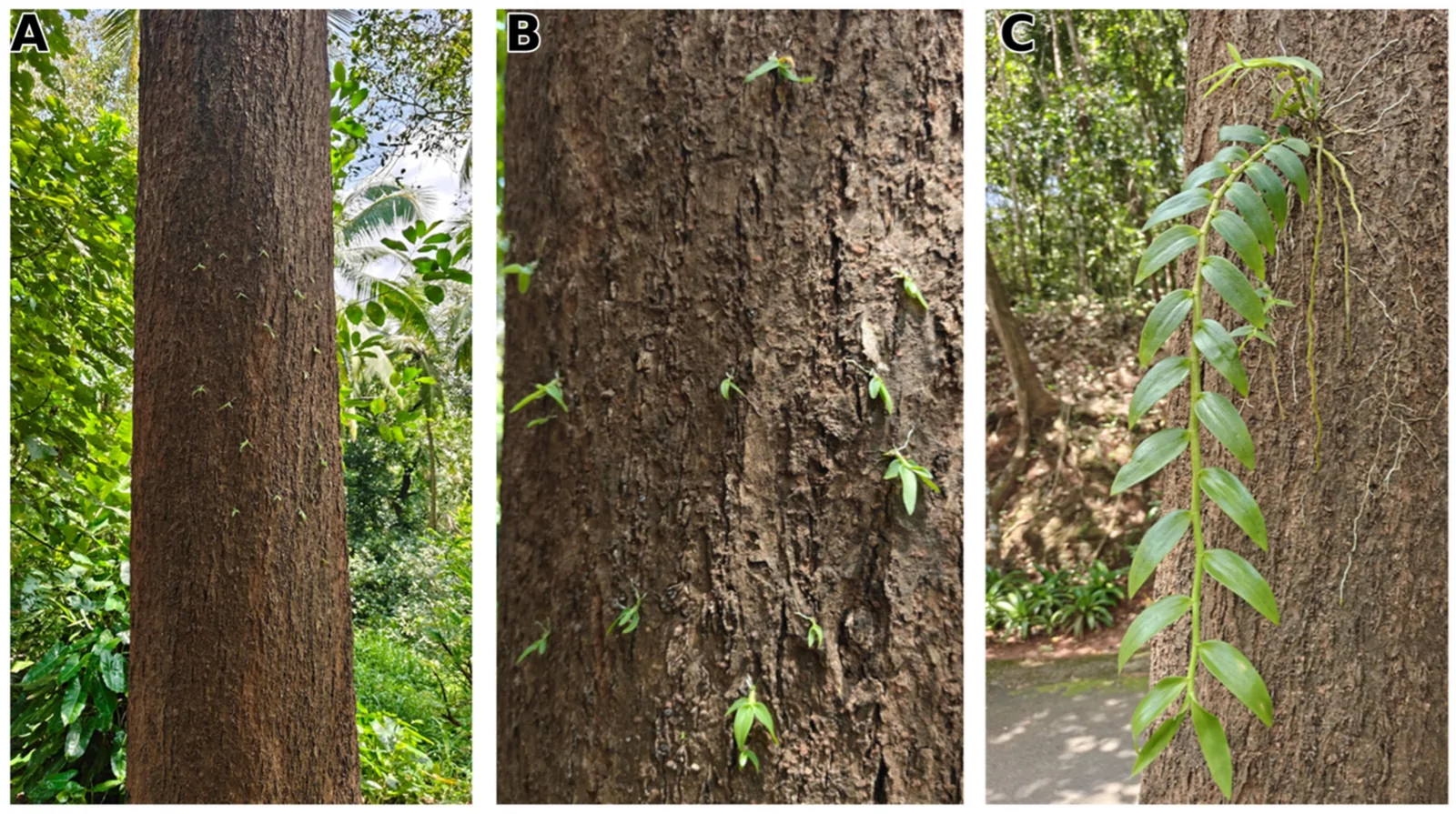
Pilot field sowing of *Dendrobium macrostachyum* on *Terminalia chebula* at Malabar Botanical Garden. (**A**) Seedlings distributed across the trunk following application of seeds mixed with leaf mould to crevices in the rugose bark. (**B**) Close view of seedlings developing leaves and attached roots within bark crevices. (**C**) An established adult plant of *D. macrostachyum* shown for reference. The seed batch was obtained from previously established plants and did not originate from the current translocated cohort.

Capsules were collected from all orchids that produced them, and assessment of full seeds (those containing embryos) confirmed viability in every capsule collection. The seeds are being stored at Malabar Botanical Garden for future full viability assessments, in vitro studies and sowing on selected phorophytes. Separately, a single-species field sowing demonstration was conducted using *Dendrobium macrostachyum* (the species failed to flower in the current planting cohort) seeds collected from previously established plants at Malabar Botanical Garden. Seeds applied to the rugose bark of the native phorophyte *Terminalia chebula* germinated, and seedlings emerged across the trunk surface. Some seedlings subsequently developed leaves and attached roots, demonstrating the feasibility of the field sowing method for early seedling establishment, but not recruitment from seeds produced by the currently monitored orchids (Figure 2).

### 2.10. Public Engagement

**Figure 3:**
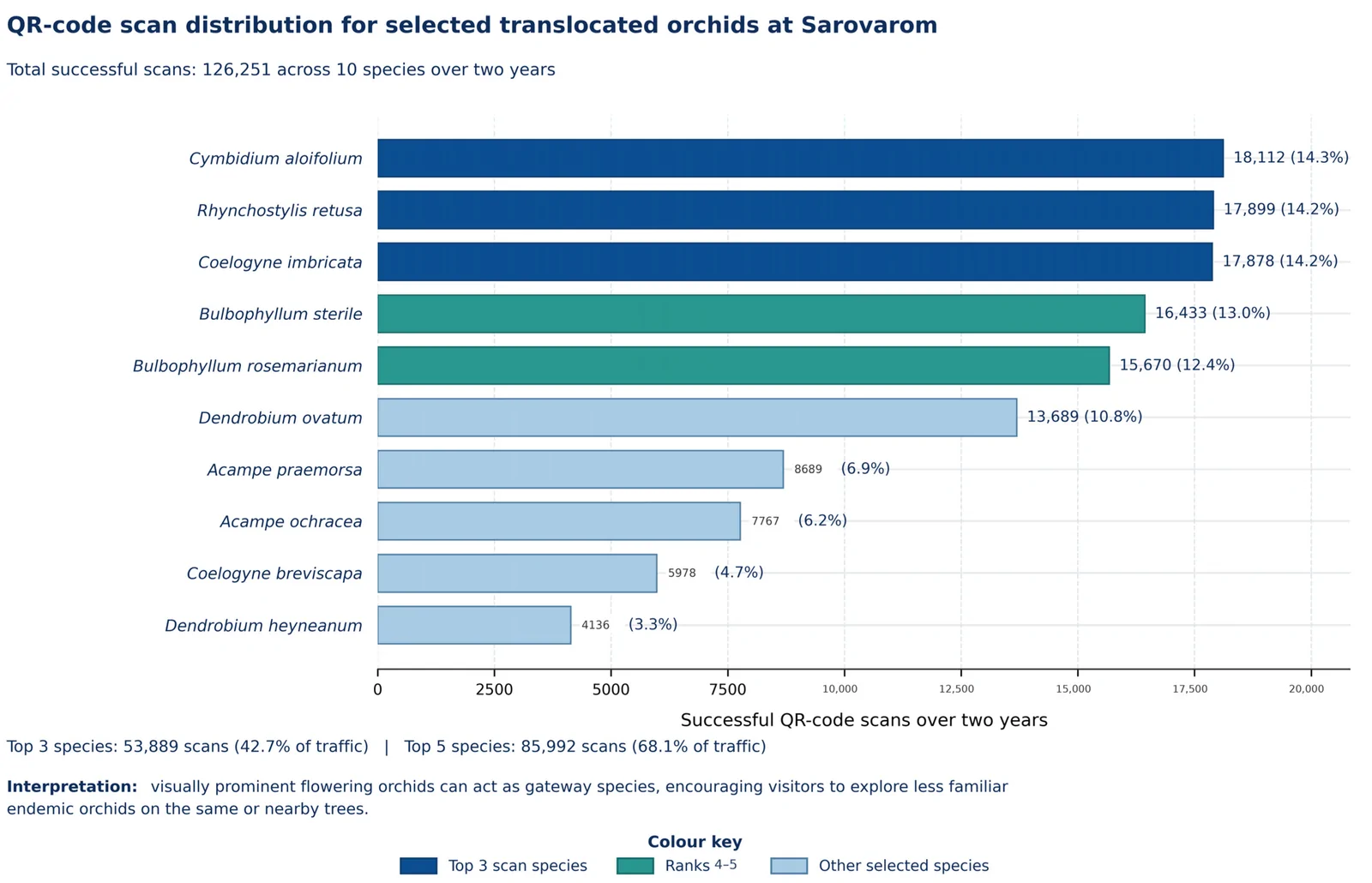
QR-code scan distribution for selected translocated orchids at Sarovaram. Values show scan totals and shares of overall QR-code traffic.

Sarovaram receives approximately 510,920 fee-paying visitors annually. Ten selected orchid labels generated 126,251 QR-code scans over 2 years. *Cymbidium aloifolium*, *Rhynchostylis retusa* and *Coelogyne imbricata* together accounted for 42.7% of QR traffic (Table 8; Figure 3). Both *Bulbophyllum* species received high numbers of scans despite lacking large, visually conspicuous flowers, suggesting that novelty and unfamiliar morphology also stimulated visitor curiosity.

**Figure 4:**
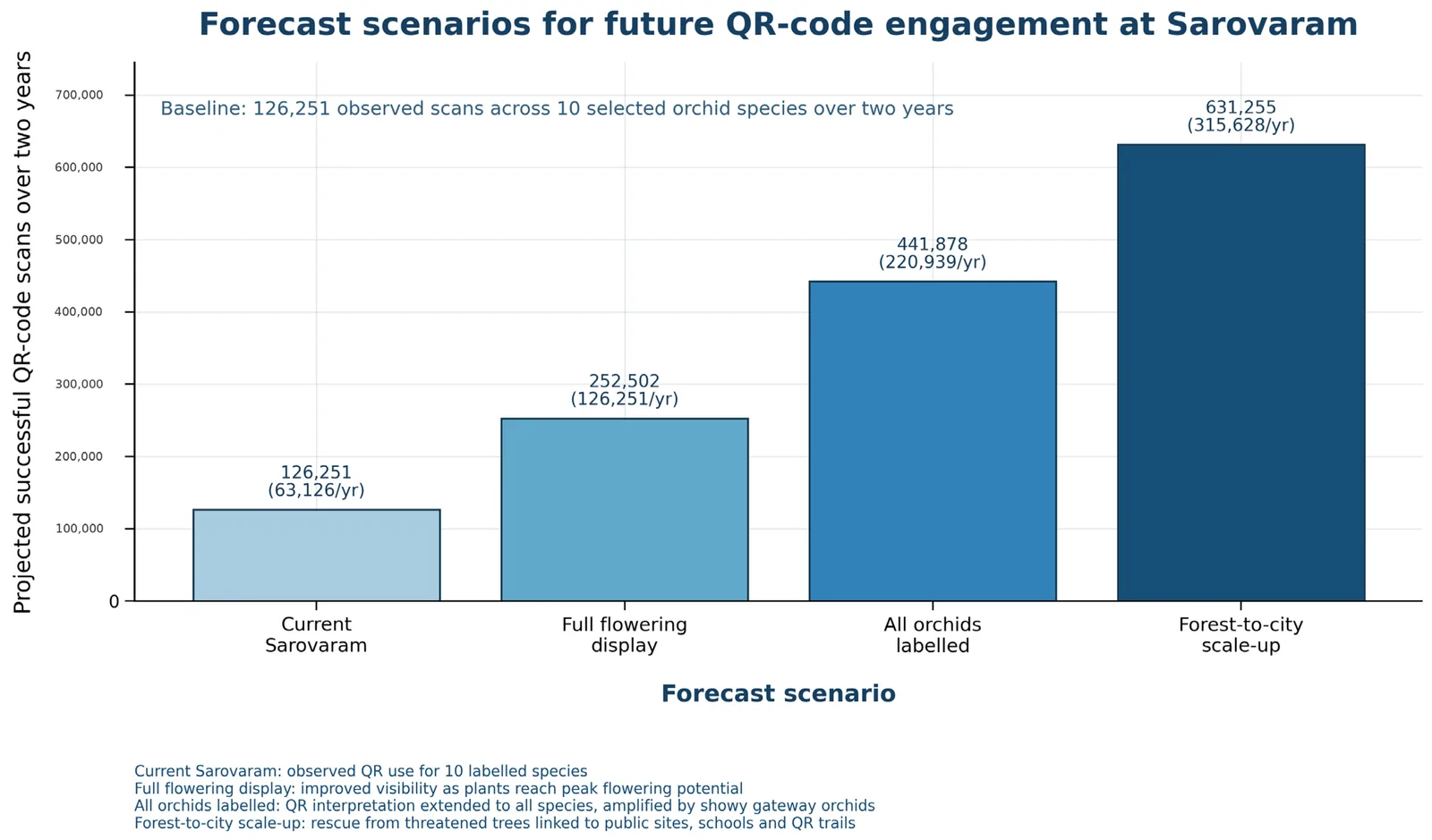
Illustrative forecast scenarios for future QR-code engagement at Sarovaram under increased flowering, labelling and interpretation.

Scenario-based projections indicated that QR-code engagement could increase with fuller flowering displays, expansion of QR-code labelling to all orchid species and wider application of the interpretation model. The projected 2-year interactions increased from the observed baseline of 126,251 to approximately 252,500 under full flowering, 441,900 with comprehensive labelling and 631,300 under forest-to-city scale-up (Figure 4). These projections are illustrative and represent potential engagement under progressively enhanced display and interpretation scenarios rather than statistically fitted forecasts.

QR-code scans demonstrated that visitors accessed the interpretive material associated with the orchid displays, providing a measure of public interaction and the reach of the information provided. These data do not directly measure knowledge acquisition, attitudinal change or subsequent conservation behaviour.

## 3. Discussion

The present orchid rescue programme responds to development-driven species loss within the wider global biodiversity crisis. In biodiversity hotspots, exceptional endemism and extensive historical habitat loss mean that even relatively small-scale development can have disproportionate and potentially irreversible consequences. Development in such regions therefore requires particularly rigorous, evidence-based mitigation, as many affected species have restricted distributions and few suitable opportunities for habitat replacement or relocation [1]. Land-use change remains the dominant direct driver of recent global biodiversity loss, emphasising the need to integrate species-level safeguards into infrastructure and urban development planning [3]. Human pressure is disproportionately intense and rapidly increasing in regions of high biodiversity, reinforcing the need for conservation safeguards to be incorporated before infrastructure decisions become irreversible [22]. Although anthropogenic biodiversity loss is accelerating, targeted conservation can halt or reverse declines where interventions are evidence-based, adequately monitored and translated into policy [4]. Previous studies have demonstrated the value of large-scale native orchid planting using in vitro-raised plants, particularly in Singapore and South Florida, where programmes led by Singapore Botanical Gardens and Fairchild Tropical Botanic Garden have supported orchid conservation and public engagement [20,21]. The present study differs by focusing on the rapid rescue of naturally established orchids from trees scheduled for removal. Since many of these rescued orchids were mature and already capable of flowering, the approach not only prevented immediate plant loss but also allowed assessment of whether translocation influenced flowering behaviour, reproductive output and seasonal flowering patterns.

### 3.1. Rescue Translocation Converted Probable Biodiversity Loss into Living Conservation Assets

The programme retained thousands of orchids from felled trees or trees scheduled for removal, where complete mortality was the most likely alternative. Many rescued orchids subsequently rooted, grew, flowered and produced capsules in public landscapes, transforming development-threatened plants into monitored assets for conservation, research and public engagement. Although rescue cannot replace habitat protection, it can prevent otherwise unavoidable mortality when development cannot retain orchid-bearing trees, an important consideration given that land-use change is a dominant driver of global biodiversity loss [3,4]. Similarly, orchid rescue surveys in logged forests in Peninsular Malaysia recovered 109 species, including rare, endemic and previously undescribed taxa, showing that trees scheduled for clearance may support substantial undocumented biological value [11].

### 3.2. Recipient Landscapes Provided Contrasting Evidence for Conservation Practice

Although the three recipient sites shared the same warm, humid and strongly monsoonal regional climate, they represented contrasting ecological environments. Kappad was an exposed maritime site where wind, direct radiation, marine aerosol and rapid drying of phorophyte surfaces may have imposed a strong establishment filter. Sarovaram Bio Park was a sheltered urban wetland in which mangroves, canals and open water may have maintained humid, thermally buffered conditions and prolonged surface moisture. Malabar Botanical Garden comprised a heterogeneous mosaic of marshy, shaded, open and moderately elevated microsites, making orchid performance more dependent on individual phorophytes and planting positions. These contrasts suggest that local exposure, canopy structure and moisture retention may have influenced establishment more strongly than regional climate or altitude. However, because meteorological conditions were recorded only at the nearby CWRDM station, these mechanisms should be regarded as ecologically plausible interpretations rather than directly measured causes.

Recipient site remained strongly associated with survival after accounting for orchid species and phorophyte identity, indicating that site-level differences were not driven solely by unequal species allocation. The high establishment at Sarovaram supports prioritising sheltered, structurally complex environments for sensitive orchids, whereas the low initial survival at Kappad indicates that exposed coastal sites require small, replicated pilot plantings before wider use. In heterogeneous landscapes such as Malabar Botanical Garden, suitability should be assessed at the level of individual phorophytes and attachment positions. Survival, flowering and capsule production should be evaluated as sequential recovery stages, with expansion guided by survival, active root attachment and performance through a climatically stressful period. Proposed effects of wind, marine aerosol, canopy buffering and bark moisture retention remain hypotheses requiring direct measurement.

The sites should therefore be viewed as a complementary conservation network rather than ranked by survival alone. This interpretation is consistent with evidence that epiphytic orchid translocation depends on species-specific requirements, host tree identity, humidity and fine-scale site conditions [20,23]. Because suitability may not be predicted reliably from broad habitat categories, conservation programmes should combine secure recipient sites with monitored experimental placements, microclimatic assessment and adaptive management [24,25]. Managed and public landscapes can thereby provide complementary habitats within biodiversity-sensitive planning [5].

### 3.3. Regional Endemic and Cool-Growing Orchids Require Cautious Recipient Site Screening

The regional endemic group comprised eight strict Indian endemics and four species restricted to India and Sri Lanka. These 12 conservation priority species showed substantial recovery, although establishment varied markedly among sites. Sarovaram supported the highest survival, Malabar Botanical Garden the highest capsule production transition, and Kappad the lowest establishment. The comparatively high flowering rate among Kappad survivors should not be interpreted as broad suitability because it was based on only 42 plants.

The subset associated with high-altitude, hill and mist forests also showed measurable recovery, with 1671 of 3146 planted individuals surviving. However, the strong site variation indicates that sensitive orchids require humid, shaded and thermally buffered recipient environments. This is consistent with evidence that rainfall, temperature and seasonal moisture influence growth, flowering and translocation success in montane orchids [26], and that epiphytic orchid performance varies with site, phorophyte and humidity [20,23]. Sarovaram likely provided greater microclimatic buffering, whereas Kappad represented a more exposed and novel environment. Coastal placements should therefore be limited to small, replicated and closely monitored trials [24,25]. Long-term recovery must also account for pollinators and compatible mycorrhizal fungi because survival and capsule production alone do not demonstrate natural recruitment or population self-sustenance [27,28,29].

### 3.4. Phorophyte Identity Was Associated Primarily with Establishment

Phorophyte identity was most strongly associated with orchid establishment, whereas its contribution to flowering among survivors was weaker and the estimated phorophyte variance for capsule production after flowering approached zero. Some frequently used phorophytes showed more positive adjusted survival estimates than others, but these estimates should not be interpreted as universal orchid–phorophyte compatibility rankings. Allocation was operational, planting numbers were unequal, and most orchid species were not replicated across the same phorophytes and recipient sites. Apparent phorophyte associations may also incorporate unmeasured variation in bark texture, moisture retention, attachment height, orientation, canopy position and local microclimate. The present results therefore support screening and monitoring multiple candidate phorophytes during early establishment rather than prescribing fixed species–phorophyte matches. Robust matching recommendations will require balanced, replicated trials in which the same orchid species is allocated across multiple phorophyte species within a common recipient environment. Accordingly, these findings support wider recommendations that orchid translocation planning and evaluation should incorporate species ecology, recipient habitat quality, pollinator availability, mycorrhizal associations and demographic monitoring, rather than relying on planting numbers alone [27,29,30].

### 3.5. Root Establishment Provides a Practical Early-Warning Indicator

Root growth was the only measured trait positively associated with all three recovery stages, making early attachment and root extension practical indicators of orchid–phorophyte suitability. Monitoring should therefore progress from root establishment to survival, flowering and capsule production, allowing poorly performing orchid–phorophyte combinations to be repositioned before irreversible decline. This staged assessment is particularly important because flowering does not guarantee pollination, and viable seeds require suitable microsites and compatible mycorrhizal fungi [30]. Evidence from plant reintroductions further shows that establishment, reproduction and next-generation recruitment represent distinct measures of success and may be governed by different factors [31,32,33].

### 3.6. Embryo-Containing Seeds and Pilot Field Establishment

The detection of embryo-containing seeds in the assessed capsule collections indicates that at least some translocated orchids progressed from flowering to potentially viable seed production. Because *Dendrobium macrostachyum* has not yet flowered within the current translocated cohort, a separate field sowing demonstration using seeds from previously established plants provided a practical opportunity to evaluate the method without waiting for the monitored plants to produce capsules. Seedling emergence and the subsequent development of leaves and attached roots indicated that rugose bark crevices on *Terminalia chebula* could support early establishment under the conditions tested. However, because the seed batch came from previously established plants and only one orchid species and phorophyte context were examined, this result demonstrates methodological feasibility rather than natural recruitment or next-generation recovery from the current translocation. Future replicated trials should use seeds produced by monitored orchids, compare phorophytes and microsites, and determine whether seedlings survive to reproductive maturity. Recruitment, rather than adult persistence alone, is required for population renewal [28,30].

### 3.7. Implications for Development Mitigation and Conservation Planning

The findings support an orchid-focused mitigation pathway that includes pre-felling surveys, rescue before unavoidable tree removal, ecological matching of species with recipient microclimates and phorophytes, and monitoring from root establishment through flowering, capsule production and recruitment. Evidence that orchid survival varies with species-specific requirements, host identity and humidity further supports carefully monitored pilot planting rather than uniform relocation [20,23]. Native tree planting should create diverse, long-lived phorophyte networks capable of remaining suitable under changing climatic conditions [34]. Periodic taxonomic review is also essential, as corrections such as *Vanda sathishii* and *Dendrobium nodosum* can alter conservation priorities. Together, these measures shift mitigation from short-term plant relocation towards rebuilding the conditions required for self-sustaining orchid populations. This rescue framework is also consistent with current IUCN guidance for taxa displaced by development, which emphasises structured assessment, risk evaluation, recipient site selection, monitoring and adaptive management [35].

### 3.8. Public Landscapes Can Connect Recovery with Engagement

QR-code scans at Sarovaram demonstrated that visitors accessed the digital interpretive material associated with the orchid displays [36]. Scan counts indicate digital interaction rather than confirmed learning, increased biodiversity knowledge or subsequent conservation behaviour. Charismatic flowering species attracted high scan numbers and may act as gateway taxa, but the substantial engagement with the small-flowered Bulbophyllum sterile indicates that novelty and unfamiliar morphology may also stimulate visitor interest. The high scan count for the less showy *Bulbophyllum sterile* suggests that visitor interest may have been influenced by novelty and unfamiliarity, not floral attractiveness alone. Small, rarely encountered endemic orchids may stimulate curiosity when made visible and identifiable through well-designed digital and on-site interpretation [36,37].

Public access to the QR-coded material indicates that accessible landscapes can support interaction with orchid conservation information. However, scan counts measure engagement with the interpretive resource rather than educational or behavioural outcomes. Demonstrating increased knowledge, changed attitudes or conservation action would require complementary visitor evaluation, such as brief pre- and post-engagement questionnaires, information recall questions or follow-up surveys [38]. Although QR traffic does not represent unique visitors or confirm learning, it provides a useful indicator of active engagement. Public landscapes can therefore combine biodiversity conservation with opportunities for education, wellbeing and nature connection when ecological planting is supported by accessible interpretation [39,40,41]. Future analyses should link species-specific scans with flowering cycles and visitor surveys, and could incorporate citizen-science recording of flowering and capsule production, to determine whether initial curiosity develops into knowledge, sustained engagement and conservation action [42,43]. In situ seed germination could transform suitable phorophytes into diverse, high-impact native orchid displays. By combining charismatic orchids with regional endemic and less conspicuous species, these living displays could increase public engagement and strengthen connections with native biodiversity.

### 3.9. Conservation Significance

The rescue translocation programme demonstrates that development-threatened orchids can be retained as living conservation assets when emergency rescue is coupled with evidence-based recipient site selection, phorophyte matching and staged monitoring. The strong establishment at Sarovaram, intermediate performance at Malabar Botanical Garden and selective filtering at Kappad show that public landscapes can provide complementary conservation functions. The recovery of 12 regional endemic species, including eight strict Indian endemics, strengthens the conservation value of the programme while emphasising that restricted-range and microclimatically sensitive orchids require particularly cautious placement and long-term stewardship. The use of public landscapes is consistent with the wider recognition that biodiversity conservation must extend beyond protected areas. Properly managed landscapes can provide supplementary habitat, ecological connectivity and public value, although these functions should complement rather than justify the loss of natural habitat [5,32,33]. The lack of global IUCN Red List assessments for 11 of the 12 regional endemic orchids (Table S2) reveals a substantial knowledge gap concerning their distribution, population abundance and trends within their restricted ranges, limiting the reliable evaluation of extinction risk and the prioritisation of conservation action.

### 3.10. Limitations and Future Monitoring

The Keralam rescue translocation programme demonstrates how development-threatened orchids can be retained as living, monitored conservation assets, but several limitations constrain interpretation. Outcomes were assessed over 12–24 months and therefore represent interim establishment and reproductive recovery rather than long-term population persistence. Orchid allocation among sites and phorophytes was operational rather than balanced or randomised, and some species × site × phorophyte combinations had small or zero denominators. Although adjusted analyses separated the contributions of orchid identity, recipient site and phorophyte, residual confounding among these factors remains possible. Regional weather was characterised using a nearby meteorological station, but microclimate was not measured at individual attachment points; explanations involving coastal exposure, canopy buffering, salt deposition and bark moisture retention therefore remain hypotheses. The separate *Dendrobium macrostachyum* field sowing demonstration used seeds from previously established plants rather than the current translocated cohort and showed methodological feasibility, not recruitment from the monitored planting. Similarly, QR-code scans measured public interaction with the interpretive material rather than learning or behavioural change. Long-term evaluation will require individual-level tracking and repeated annual censuses for at least 5–10 years, direct microclimate measurements, pollinator and mycorrhizal assessments, seed viability studies, structured visitor surveys, and monitoring of whether recruited seedlings survive, mature and reproduce. Species-specific phorophyte suitability should be tested using balanced within-site trials with replicated trees and comparable planting numbers.

## 4. Conclusions

This study demonstrates that rescue translocation can transform orchids facing otherwise inevitable destruction during infrastructure development into living conservation assets. Recovery across three contrasting landscapes confirmed that mature epiphytic orchids can re-establish, flower and produce capsules containing embryo-bearing seeds when placed in suitable recipient environments.

The sites provided complementary conservation functions: Sarovaram supported establishment, Malabar Botanical Garden enabled orchid–phorophyte evaluation and seed conservation, and Kappad tested tolerance under coastal exposure. Phorophyte identity was most influential during establishment, while root growth emerged as a practical early indicator of recovery.

The interim recovery of regional endemic and cool-growing orchids is particularly important because many have restricted distributions and remain unevaluated for the IUCN Red List. Seeds from previously established *Dendrobium macrostachyum* plants allowed the feasibility of field seed baiting to be assessed without waiting for the current translocated cohort to flower and produce capsules. Seedling emergence and the development of leaves and attached roots indicated that the selected phorophyte could support early establishment. However, as the seed batch did not originate from the current cohort and only one species was tested, the result demonstrates the potential of the method rather than natural recruitment or next-generation recovery. Future replicated trials should use seeds produced by monitored plants and assess whether seedlings survive to reproductive maturity. Given the naturally low and episodic recruitment of many epiphytic orchids, monitoring will continue over 5–10 years to assess flowering, viable seed set, and seedling recruitment and establishment.

Future assessments should integrate anonymised QR-code analytics with structured visitor surveys to distinguish audience reach from changes in conservation knowledge, attitudes and intended or reported behaviour. Both charismatic and inconspicuous orchids stimulated curiosity, demonstrating the value of presenting diverse native species together. Greater impact could be achieved through in situ seed germination to establish dense, diverse displays of charismatic and regional endemic orchids on suitable phorophytes. These living displays could increase public engagement and strengthen people’s connection with Keralam’s largely unfamiliar native biodiversity.

Overall, the Keralam programme provides a practical framework for biodiversity-sensitive development. By integrating pre-felling surveys, timely rescue, ecological matching, native phorophyte planting, long-term monitoring and public interpretation, this model can reduce avoidable orchid loss and support species recovery across the Western Ghats and other orchid-rich regions. Species-specific phorophyte suitability remains a priority for future study using balanced, replicated comparisons of the same orchid species across multiple phorophytes within common recipient environments.

## 5. Materials and Methods

### 5.1. Study Area and Recipient Sites

The study was conducted in Kozhikode, Keralam, India, across three recipient landscapes: Sarovaram Biopark (2–14 m a.s.l), Malabar Botanical Garden (10–30 m a.s.l.) and Kappad Beach (0–6 m a.s.l.). Kappad is likely the most exposed of the three sites. Its proximity to the Arabian Sea means that orchids experience stronger air movement, salt-laden winds, intense solar exposure and rapid changes associated with monsoon weather. Sarovaram is a low-lying urban wetland associated with mangroves, canals and standing water. These features may maintain relatively high local humidity, reduce daytime heating and prolong surface moisture on shaded trunks and roots. The site contains approximately 200 acres of protected wetland and mangrove habitat adjacent to the Canoly Canal. Malabar Botanical Garden was the most spatially heterogeneous of the three sites. The garden includes substantial marshy land as well as moderately elevated or hilly terrain, producing variation in soil moisture, canopy cover, shade, air movement and drainage over relatively short distances (Figure S1, Table S1). All three recipient sites were situated within the low-elevation coastal plain of Kozhikode, although their elevations and topographic settings differed. Kappad was a near-sea-level coastal site, Sarovaram Bio Park occupied a low-lying urban wetland, and Malabar Botanical Garden comprised a heterogeneous landscape of marshy lowland and moderately elevated terrain.

### 5.2. Rescue Translocation Procedure

Orchids were rescued from trees that had already been felled or were scheduled for felling during road expansion. Because this was an emergency conservation operation rather than a prospectively designed experiment, rescue and placement were undertaken within the time available before or immediately after tree removal. Rescued clumps were identified, their condition was assessed, and they were transported to one of the three available recipient landscapes. Allocation among recipient sites, phorophytes and attachment positions was not randomised or balanced. Orchid identity, field experience and apparent sensitivity to exposure or desiccation informed placement where practicable; however, detailed source-site microclimate and bark characteristics were not measured systematically. Actual allocation was also strongly influenced by rescue timing, the number and size of clumps recovered, transport logistics, suitable phorophyte availability, accessibility for attachment, and capacity for subsequent care and monitoring. Within recipient sites, phorophytes were selected on the basis of apparent tree health, physical stability, available attachment area and the suitability of the bark and canopy position for securing the clumps. Attachment positions were chosen to provide stable bark contact and, where practicable, shade and shelter from direct exposure. Bark properties, attachment height, orientation, canopy openness and microclimate were not recorded consistently during the emergency operation. Consequently, the recorded orchid species × recipient site × phorophyte combinations represent realised operational allocations rather than a balanced factorial test of orchid–phorophyte compatibility.

### 5.3. Dataset and Response Variables

Translocation outcomes were assessed over 12–24 months, encompassing at least one complete annual flowering cycle and the expected acclimatisation and flowering periods of all monitored species. Recovery was evaluated sequentially as early establishment, based on survival and active root growth or attachment; continued survival during subsequent assessments; and reproduction, based on flowering and capsule production. Capsule collections were examined for seeds containing visible embryos, and field establishment was documented for a single species, *Dendrobium macrostachyum*, in a separate pilot-sowing demonstration. Derived response variables were survival among planted individuals, flowering among planted individuals, capsule production among planted individuals, flowering among surviving plants, and capsule production among flowering plants.

### 5.4. Seed Assessment, Storage and Field Recruitment Trial

Mature capsules were collected from orchids that produced capsules, and seeds were examined for the presence of embryos before storage. Embryo-containing seed collections were retained at Malabar Botanical Garden for future conservation research. A separate pilot field sowing trial was conducted using *Dendrobium macrostachyum* seeds sourced from plants established during an earlier rescue trial at Malabar Botanical Garden. This seed source was used because only a very small number of plants survived in the current trial and none produced flowers. Seeds mixed with leaf mould were applied to crevices in the rugose bark of *Terminalia chebula*. Germination, seedling emergence, leaf development and root attachment were monitored photographically. The trial was intended to demonstrate the feasibility of the field sowing method rather than assess natural recruitment from the current planting.

### 5.5. Regional Endemic Classification and Accepted Name Changes

Two related but distinct endemism variables were retained after accepted name reconciliation. Strict_India_endemic was coded “Yes” only for species whose accepted native range was restricted to India. India_Sri_Lanka_regional_endemic was coded “Yes” for species whose accepted native range was restricted to India and Sri Lanka. Regional_endemic_analysis_group, the binary variable used in the statistical models, was coded “Yes” for both groups and “No” for all other native orchid species. The combined analytical group therefore comprised 12 species: eight strict Indian endemics and four India–Sri Lanka shared regional endemics. Accepted names and native distributions were verified using Plants of the World Online and the World Checklist of Vascular Plants. The classification was joined to the outcome dataset by accepted orchid name before model fitting.

The 12 regional endemic taxa, accepted name treatments, native distributions and global IUCN Red List status are summarised in Table S4. Table S2 now reports Strict_India_endemic, India_Sri_Lanka_regional_endemic, Regional_endemic_analysis_group, Biogeographic_category, Accepted_name_used, and Original_dataset_name as separate fields. Not Evaluated indicates the absence of a global assessment and should not be interpreted as evidence of low conservation risk.

*Flickingeria nodosa* was mapped to the accepted name *Dendrobium nodosum*. The Keralam material originally recorded as *Vanda thwaitesii* was reassigned to Vanda sathishii; this was treated as a correction of the dataset identification rather than a formal synonym relationship. Every outcome row belonging to a given accepted species was assigned the same endemism classification.

### 5.6. “Connecting People with Nature” Metrics

Sarovaram monthly paid visitor numbers were compared with orchid flowering seasonality to identify periods when public footfall and orchid display value overlapped most strongly. To assess public engagement with the translocated orchids at Sarovaram, QR-code traffic data were analysed for 10 selected orchid species over a 2-year period. For each species, the total number of QR-code scans was extracted from the QR-code dataset. Species-level scan totals were ranked to identify the orchids generating the highest visitor interaction. The proportion of total QR-code traffic contributed by each species was calculated as: species scan share = species scans/total scans × 100.

The top three and top five scan-generating species were summarised to assess whether a small number of charismatic or visually prominent orchids acted as public engagement “gateway species”. QR-code scan totals were interpreted in relation to annual Sarovaram visitation, where paid entry has exceeded half a million visitors annually, in addition to daily unpaid use by walkers and people exercising. The projections were scenario-based rather than a formal time-series model, because the available QR dataset contained total scans per species over 2 years, but not monthly scan counts linked directly to flowering intensity. Future engagement was projected using simple multiplier scenarios based on expected increases in visibility, full flowering, expanded QR labelling and active interpretation.

### 5.7. Statistical Analysis

Analyses were conducted using the original count data, with each site × orchid species × phorophyte combination treated as the analytical unit. Site-level differences in survival, flowering and capsule production were initially assessed using contingency-table χ^2^ tests. Three binomial generalised linear mixed models (GLMMs) with logit links were then fitted to evaluate successive stages of recovery: (i) survival among planted orchids, modelled as survived versus not survived; (ii) flowering among surviving plants, modelled as flowered versus not flowered; and (iii) capsule production among flowering orchids, modelled as capsule-producing versus non-capsule-producing. Records with a zero denominator were excluded only from the relevant model. Recipient site, regional endemic status and their interaction were included as fixed effects, with orchid species and phorophyte identity included as crossed random intercepts. Regional endemic status was joined by accepted orchid name and coded 1 for the 12 species in the combined regional endemic analysis group and 0 for all other native species. Because allocation was operational and unbalanced, site, orchid species and phorophyte terms were interpreted as adjusted observational associations rather than causal effects arising from randomised assignments.

Population-level predicted probabilities and 95% confidence intervals were calculated for each recipient site x regional endemic-status combination from the fixed-effect linear predictors, with orchid species and phorophyte random intercepts set to their mean of zero. This represents an adjusted average orchid and phorophyte rather than the observed proportion or a prediction conditional on a named species–phorophyte combination. The corrected 12-species Regional_endemic_analysis_group was used in all three models.

Model adequacy was assessed using optimisation convergence, residual gradients, random effect variances, residual dispersion and confidence interval widths. The survival model reported numerical precision loss despite a very small residual gradient and was interpreted cautiously; the flowering model converged successfully; and the capsule production model remained non-convergent and was treated as exploratory. Fixed effects are reported as odds ratios with 95% confidence intervals and two-sided Wald *p*-values, with statistical significance assessed at *p* < 0.05. Analyses were conducted in Python 3.12.14 using statsmodels 0.15.0, pandas 2.2.2 and NumPy 1.26.4; figures were prepared using Matplotlib 3.8.4.

## Figures and Tables

**Table 1:** Site-level establishment and reproductive performance of all translocated orchids.

Site	Planted	Survived	Flowered	Capsule-Producing	Survival (%)	Flowered/Planted (%)	Capsules/Planted (%)
Sarovaram	17,381	15,244	4013	1395	87.7	23.1	8.0
Malabar Botanical Garden	7601	3595	972	390	47.3	12.8	5.1
Kappad Beach	2342	672	239	149	28.7	10.2	6.4

Values are observed counts and percentages. Survival = survived/planted; flowered/planted = flowered/planted; capsules/planted = capsule-producing/planted.

**Table 2:** Chi-square tests comparing site-level differences in orchid translocation outcomes.

Response	Chi-Square	df	*p*-Value
Survival	6517.98	2	<0.001
Flowering among planted orchids	494.48	2	<0.001
Capsule production among planted orchids	69.39	2	<0.001
Flowering among survivors	28.19	2	<0.001
Capsule production among flowering orchids	78.52	2	<0.001

Chi-square tests compared aggregate proportions among recipient sites.

**Table 3:** Likelihood ratio tests of independent predictors of orchid translocation survival.

Effect	LR Chi-Square	df	*p*-Value	Statistical Outcome
Site	881.61	2	<0.001	Significant
Species	3806.13	20	<0.001	Significant
Phorophyte	985.98	25	<0.001	Significant

**Table 4:** Effects of growth traits on orchid recovery.

Outcome	Predictor	OR per SD	95% CI	*p*-Value
Survival	Root growth	1.74	1.30–2.33	<0.001
Survival	Leaf length	4.59	2.95–7.15	<0.001
Survival	Leaf width	0.8	0.65–0.98	0.029
Flowering among survivors	Root growth	1.73	1.31–2.29	<0.001
Flowering among survivors	Leaf length	0.28	0.22–0.35	<0.001
Flowering among survivors	Leaf width	1.67	1.38–2.02	<0.001
Capsule production among flowering orchids	Root growth	1.67	1.05–2.66	0.029
Capsule production among flowering orchids	Leaf length	0.23	0.14–0.35	<0.001
Capsule production among flowering orchids	Leaf width	1.17	0.81–1.68	0.406

Odds ratios are per standard deviation increase; models adjusted for site, orchid species and phorophyte identity.

**Table 5:** Observed establishment and reproductive performance of regional endemic orchids.

Site	Planted	Survived	Flowered	Capsule-Producing	Survival (%)	Flowering/Survivors (%)	Capsules/Flowering (%)
Sarovaram	3997	2503	366	146	62.6	14.6	39.9
Malabar Botanical Garden	1813	823	130	57	45.4	15.8	43.8
Kappad Beach	316	42	14	4	13.3	33.3	28.6
Total	6126	3368	510	207	55.0	15.1	40.6

**Table 6:** Observed recovery of high-altitude, hill- and mist forest-associated regional endemic orchids.

Site	Planted	Survived	Flowered	Capsule-Producing	Survival (%)	Flowering/Survivors (%)	Capsules/Flowering (%)
Sarovaram	2542	1432	254	80	56.3	17.7	31.5
Malabar Botanical Garden	474	218	85	33	46.0	39.0	38.8
Kappad Beach	130	21	10	3	16.2	47.6	30.0
Total	3146	1671	349	116	53.1	20.9	33.2

The subset comprised *Coelogyne breviscapa*, *Dendrobium heyneanum*, *D. barbatulum*, *Bulbophyllum fimbriatum*, *Aerides crispa* and *D. ovatum*.

**Table 7:** Population-level predicted probabilities of survival, flowering and capsule production by recipient site and regional endemic status.

Recovery Stage	Site	Other Native, % (95% CI)	Regional Endemic, % (95% CI)
Survival	Sarovaram	78.7 (60.1–90.0)	22.7 (11.5–39.9)
	Malabar Botanical Garden	28.0 (13.7–48.8)	13.0 (6.2–25.4)
	Kappad Beach	14.4 (6.4–29.4)	2.9 (1.2–6.9)
Flowering among survivors	Sarovaram	28.0 (23.8–32.7)	15.1 (12.2–18.5)
	Malabar Botanical Garden	32.5 (27.7–37.6)	22.7 (17.9–28.4)
	Kappad Beach	40.1 (33.4–47.1)	40.3 (24.9–57.9)
Capsule production among flowering plants	Sarovaram	34.8 (31.3–38.6)	39.8 (33.7–46.2)
	Malabar Botanical Garden	40.4 (35.5–45.4)	44.0 (34.9–53.6)
	Kappad Beach	64.5 (57.0–71.4)	31.9 (13.3–58.7)

Values are population-level predicted probabilities with 95% confidence intervals from crossed binomial mixed-effects models. Flowering was modelled among surviving plants, and capsule production among flowering plants. Predictions were calculated with orchid species and phorophyte random effects set to zero. Capsule production estimates are exploratory because the model did not converge satisfactorily.

**Table 8:** Sarovaram visitation, orchid display, and public engagement opportunity.

Metric/Period	Visitor Relevance	Display/Public Value	Recommended Focus
Annual visitors	510,920/year	Large public audience	Flagship conservation landscape
QR-code scans	126,251 over 2 years	Approximately 63,126/year	Expand labels across species
March	High relevance	Increasing flowering	Start-of-season interpretation
April	Strongest overlap	Peak display	Main orchid-awareness month
May	Continued high use	Flowering remains important	Capsule development
September	Good off-season relevance	High species richness	Regional endemic diversity

Scans indicate successful interactions, not unique visitors.

## Data Availability

The original contributions presented in this study are included in the Supplementary Material. Further inquiries can be directed to the corresponding author.

## Supplementary Materials

The following supporting information can be downloaded at: https://www.mdpi.com/article/10.3390/plants15203153/s1, Figure S1. Study areas in Kozhikode, Keralam, India-A. India map showing the three sites, B. Keralam map showing the three sites, C. Kozhikode map showing the three sites; Figure S2.1. Sarovaram Biopark displaying numerous orchid species growing on diverse tree species; S2.2 (A-C)-A. Malabar Botanic Garden; B. *Cymbidium aloifolium* growing on *Syzygium cumini*; C. *Cymbidium aloifolium* growing on *Mangifera indica*; S2.3., A. Kappad Beach; B *Dendrobium macrostachyum* growing on *Pongamia pinnata*; C. *Cymbidium aloifolium* growing on *Casuarina equisetifolia*; Table S1. Regional climate and ecological characteristics of the three orchid recipient sites; Table S2. Sheets 1–3. Allocation and outcomes of rescued epiphytic orchids and model-partitioning analyses; Table S3. Operational guidance for epiphytic orchid translocation; Table S4. Taxonomic verification, regional endemic classification and conservation status; and taxonomic and distribution citations S1–S14.
