# Supplementary material for: When Roads Remove Trees, Conservation Must Move Fast: A Rescue Translocation Model for Keralam’s Native Epiphytic Orchids
Source: Plants (Basel). 2026 Oct 15;15(20):3153. doi: 10.3390/plants15203153 (PMC13709079; doi:10.3390/plants15203153)
Supplement: Supplementary file 1 [file plants-15-03153-s001.zip › Table_S1.pdf]

Table S1. Regional climate and ecological characteristics of the three orchid recipient sites

Characteristic	Kappad Beach	Sarovaram Bio Park	Malabar Botanical Garden
Ecological setting	Exposed maritime site near the Arabian Sea	Sheltered urban wetland with mangroves and open water	Heterogeneous inland garden with marshy, shaded and open habitats
Local environmental conditions	Greater wind, solar and salt-spray exposure; rapid drying of bark and aerial roots	Humid, thermally buffered and relatively sheltered; prolonged surface moisture	Variable shade, humidity and exposure among phorophytes and planting positions
Principal establishment filter	Tolerance of coastal exposure and post-rainfall drying	Tolerance of humid, shaded and occasionally persistently wet conditions	Diverse native trees as phorophytes and microsite
Regional climate	Warm, humid and strongly monsoonal	Warm, humid and strongly monsoonal	Warm, humid and strongly monsoonal
Regional weather summary	Mean annual rainfall: 3,236 mm; mean maximum/minimum temperature: 32.6/23.2°C; maximum/minimum RH: 90.6/68.6%	Same regional weather record	Same regional weather record

Note. Regional weather values were derived from daily observations at the nearby CWRDM Campus meteorological station, Kottamparamba, during 2005–2025. In the absence of separate weather stations at the recipient sites, these data characterise their shared regional climate. Local conditions reflect differences in coastal exposure, wetland influence and habitat structure and were not measured directly at individual orchid attachment points.
