# Supplementary material for: When Roads Remove Trees, Conservation Must Move Fast: A Rescue Translocation Model for Keralam’s Native Epiphytic Orchids
Source: Plants (Basel). 2026 Oct 15;15(20):3153. doi: 10.3390/plants15203153 (PMC13709079; doi:10.3390/plants15203153)
Supplement: Supplementary file 1 [file plants-15-03153-s001.zip › Table_S3.pdf]

Table S3. Operational guidance for epiphytic orchid translocation

Decision stage	Finding directly supported by this study	Interpretation or mechanism requiring testing	Operational recommendation
**Recipient-site screening**	Survival differed strongly among recipient sites and the site effect remained significant after adjustment for orchid species composition and phorophyte identity.	Differences may reflect local variation in exposure, canopy structure, humidity, bark wetness and other microsite conditions that were not measured directly.	Assess canopy openness, shelter, prevailing-wind exposure, proximity to the coast or wetlands, and availability of mature phorophytes before allocation. Do not infer suitability from regional climate or geographical proximity alone.
**Sheltered recipient environments**	Sarovaram Biopark supported the highest establishment among the three recipient sites.	Mangroves, wetland vegetation, surface water and canopy cover may have produced a more humid and thermally buffered environment.	Prioritise sheltered, partially shaded and structurally complex sites for restricted-range, cool-growing or otherwise sensitive orchids, while confirming suitability through monitoring.
**Exposed coastal environments**	Kappad Beach had the lowest initial survival, although some surviving plants subsequently flowered and produced capsules.	Wind exposure, direct radiation, marine aerosol and rapid post-rainfall drying are plausible causes of establishment failure but were not measured directly.	Treat exposed coastal sites as higher-risk environments. Begin with small, replicated pilot plantings across sheltered and exposed positions, and expand only after successful survival and active root attachment through a stressful climatic period.
**Heterogeneous recipient landscapes**	Performance at Malabar Botanical Garden varied among orchid-phorophyte combinations.	Variation may reflect differences among shaded, marsh-adjacent, open and elevated microsites within the garden.	Evaluate botanical gardens and similar landscapes at the individual-phorophyte and attachment-position scale rather than treating the entire landscape as environmentally uniform.
**Species allocation**	Orchid identity made the largest adjusted contribution to survival, flowering and capsule production.	Restricted-range and hill- or mist-forest-associated species may be particularly sensitive to departures from their source microclimate.	Classify species provisionally by ecological sensitivity; allocate sensitive taxa first to buffered sites and distribute irreplaceable material across multiple suitable phorophytes or recipient sites. Treat resulting associations as provisional until tested experimentally.
**Phorophyte selection**	Phorophyte identity contributed independently to recovery, with the strongest contribution during establishment; evidence for differences in capsule production after flowering was weak.	Bark texture, water retention, canopy position and attachment orientation and local microclimate may underlie these associations but were not measured consistently.	Where rescued material and recipient trees permit, distribute each orchid species across multiple candidate phorophytes rather than presuming a single preferred association. Record independent tree identity, bark characteristics, attachment height, orientation, canopy position and early root attachment.
**Monitoring and scaling**	Root growth was consistently associated with survival and reproductive recovery; survival, flowering and capsule production represented sequential recovery stages.	Short-term persistence without new attachment may overestimate successful establishment.	Use survival plus active new-root growth and attachment as the initial decision checkpoint. Assess flowering among survivors and capsule production among flowering plants separately, while retaining planted numbers when reporting overall conservation return.
**Adaptive management**	Site rankings differed between initial establishment and reproduction among survivors.	A site may filter many individuals during establishment while permitting good reproduction among the subset that survives.	Predefine pilot success criteria appropriate to the species and project, monitor across wet and dry or pre-monsoon periods, and modify the phorophyte, attachment position or recipient site when mortality remains high or root attachment is poor.
**Future testing**	The study detected adjusted site, species and phorophyte associations but did not measure microclimate at individual attachment points.	The proposed mechanisms cannot be assigned causally without direct environmental measurements.	Future trials should record temperature, relative humidity, light or canopy openness, wind exposure, bark-moisture persistence and, at coastal sites, salt deposition at orchid attachment points.
