# Supplementary material for: When Roads Remove Trees, Conservation Must Move Fast: A Rescue Translocation Model for Keralam’s Native Epiphytic Orchids
Source: Plants (Basel). 2026 Oct 15;15(20):3153. doi: 10.3390/plants15203153 (PMC13709079; doi:10.3390/plants15203153)
Supplement: Supplementary file 1 [file plants-15-03153-s001.zip › Table_S4.pdf]

Table S4. Taxonomic verification, biogeographic classification and global conservation status of orchids reviewed for the regional-endemic analysis and associated taxonomic checks.

Original dataset name	Accepted name used	Name treatment	Biogeographic category	Accepted native range	Global IUCN status	Citations
Aerides crispa	*Aerides crispa*	No change	Strict Indian endemic	Western India	Least Concern (2025)	[S1, S13]
Bulbophyllum fimbriatum	*Bulbophyllum fimbriatum*	No change	Strict Indian endemic	South-western India	Not Evaluated	[S2]
Bulbophyllum rosemarianum	*Bulbophyllum rosemarianum*	No change	Strict Indian endemic	India (Kerala)	Not Evaluated	[S3]
Dendrobium barbatulum	*Dendrobium barbatulum*	No change	Strict Indian endemic	South-western India	Not Evaluated	[S4]
Dendrobium heyneanum	*Dendrobium heyneanum*	No change	Strict Indian endemic	Southern India	Not Evaluated	[S5]
Flickingeria nodosa	*Dendrobium nodosum*	Synonym mapped to accepted name	Strict Indian endemic	Southern India	Not Evaluated	[S6]
Dendrobium ovatum	*Dendrobium ovatum*	No change	Strict Indian endemic	South-western India	Not Evaluated	[S7]
Vanda thwaitesii	*Vanda sathishii*	Dataset identification corrected; not treated as a taxonomic synonym	Strict Indian endemic	India (Kerala)	Not Evaluated	[S8]
Coelogyne breviscapa	*Coelogyne breviscapa*	No change	India–Sri Lanka shared regional endemic	Southern India and Sri Lanka	Not Evaluated	[S9]
Gastrochilus acaulis	*Gastrochilus acaulis*	No change	India–Sri Lanka shared regional endemic	India and Sri Lanka	Not Evaluated	[S10]
Luisia birchea	*Luisia birchea*	No change	India–Sri Lanka shared regional endemic	South-western India and Sri Lanka	Not Evaluated	[S11]
Vanda wightii	*Vanda wightii*	No change	India–Sri Lanka shared regional endemic	South-western India and central Sri Lanka	Not Evaluated	[S12]

Original dataset name	Accepted name used	Name treatment	Biogeographic category	Accepted native range	Global IUCN status	Citations
Pholidota imbricata	*Coelogyne imbricata*	Synonym mapped to accepted name	Widespread Asian–Pacific native	Tropical and subtropical Asia to the south-western Pacific	Not Evaluated	[S14]

Regional endemic was defined operationally as a species whose accepted native range is restricted to India, Sri Lanka or both. The analytical category therefore comprised eight strict Indian endemics and four species shared between India and Sri Lanka. Flickingeria nodosa is listed by POWO as a synonym of Dendrobium nodosum. The replacement of Vanda thwaitesii with Vanda sathishii was a correction to the identification of the Kerala material in the study dataset and should not be presented as a formal synonym relationship. Names and distributions were checked against Plants of the World Online (POWO), using the World Checklist of Vascular Plants taxonomic backbone. Global conservation status was checked against the IUCN Red List version 2026-1. Not Evaluated means that no published global IUCN Red List assessment was located under the accepted name; it does not mean that the species is not threatened. Sources accessed 23 August 2026.

Taxonomic and distribution citations

[S1] Aerides crispa Lindl. Plants of the World Online, Royal Botanical Gardens, Kew. https://powo.science.kew.org/ (accessed 23 August 2026).

[S2] *Bulbophyllum fimbriatum (Lindl.) Rchb.f.*. Plants of the World Online, Royal Botanical Gardens, Kew. https://powo.science.kew.org/taxon/urn:lsid:ipni.org:names:618931-1 (accessed 23 August 2026).

[S3] *Bulbophyllum rosemarianum C.S.Kumar, P.C.S.Kumar & Saleem*. Plants of the World Online, Royal Botanical Gardens, Kew. https://powo.science.kew.org/taxon/urn:lsid:ipni.org:names:20007240-1 (accessed 23 August 2026).

[S4] *Dendrobium barbatulum Lindl.*. Plants of the World Online, Royal Botanical Gardens, Kew. https://powo.science.kew.org/taxon/urn:lsid:ipni.org:names:626893-1 (accessed 23 August 2026).

[S5] *Dendrobium heyneanum Lindl.*. Plants of the World Online, Royal Botanical Gardens, Kew. https://powo.science.kew.org/taxon/urn:lsid:ipni.org:names:627617-1 (accessed 23 August 2026).

[S6] *Dendrobium nodosum Dalzell*. Plants of the World Online, Royal Botanical Gardens, Kew. https://powo.science.kew.org/taxon/urn:lsid:ipni.org:names:628107-1 (accessed 23 August 2026).

[S7] *Dendrobium ovatum (L.) Kraenzl.*. Plants of the World Online, Royal Botanical Gardens, Kew. https://powo.science.kew.org/taxon/urn:lsid:ipni.org:names:628169-1 (accessed 23 August 2026).

[S8] *Vanda sathishii Motes*. Plants of the World Online, Royal Botanical Gardens, Kew. https://powo.science.kew.org/taxon/urn:lsid:ipni.org:names:77299028-1 (accessed 23 August 2026).

[S9] *Coelogyne breviscapa Lindl.*. Plants of the World Online, Royal Botanical Gardens, Kew. https://powo.science.kew.org/taxon/urn:lsid:ipni.org:names:623788-1 (accessed 23 August 2026).

[S10] *Gastrochilus acaulis (Lindl.) Kuntze*. Plants of the World Online, Royal Botanical Gardens, Kew. https://powo.science.kew.org/taxon/urn:lsid:ipni.org:names:635383-1 (accessed 23 August 2026).

[S11] *Luisia birchea Blume*. Plants of the World Online, Royal Botanical Gardens, Kew. https://powo.science.kew.org/taxon/urn:lsid:ipni.org:names:641791-1 (accessed 23 August 2026).

[S12] *Vanda wightii Rchb.f.*. Plants of the World Online, Royal Botanical Gardens, Kew. https://powo.science.kew.org/taxon/urn:lsid:ipni.org:names:661102-1 (accessed 23 August 2026).

[S13] Jalal JS, Jayanthi J, Agrawala DK, Kumar P (2025) Aerides crispa. The IUCN Red List of Threatened Species 2025: e.T13417337A13417340. https://www.iucnredlist.org/species/13417337/13417340 (accessed 23 August 2026).
[S14] *Coelogyne imbricata* (Hook.) Rchb.f. Plants of the World Online. Facilitated by the Royal Botanic Gardens, Kew. Available at: https://powo.science.kew.org/taxon/urn:lsid:ipni.org:names:623892-1. Accessed 11 September 2026.
